# Uncarboxylated osteocalcin alleviates the inhibitory effect of high glucose on osteogenic differentiation of mouse bone marrow–derived mesenchymal stem cells by regulating TP63

**DOI:** 10.1186/s12860-021-00365-7

**Published:** 2021-04-27

**Authors:** Fangzi Gong, Le Gao, Luyao Ma, Guangxin Li, Jianhong Yang

**Affiliations:** 1 Medical School, University of Chinese Academy of Sciences, Beijing, China; 2 College of sports medicine and physical therapy, Beijing Sport University, Beijing, China

**Keywords:** TP63, Uncarboxylated osteocalcin, Bone marrow mesenchymal stem cell, Osteogenesis, High glucose

## Abstract

**Background:**

Progressive population aging has contributed to the increased global prevalence of diabetes and osteoporosis. Inhibition of osteogenic differentiation of bone marrow mesenchymal stem cells (BMSCs) by hyperglycemia is a potential pathogenetic mechanism of osteoporosis in diabetic patients. Uncarboxylated osteocalcin (GluOC), a protein secreted by mature osteoblasts, regulates bone development as well as glucose and lipid metabolism. In our previous studies, GluOC was shown to promote osteoblastic differentiation of BMSCs; however, the underlying mechanisms are not well characterized. Tumor protein 63 (TP63), as a  transcription factor, is closely related to bone development and glucose metabolism.

**Results:**

In this study, we verified that high glucose suppressed osteogenesis and upregulated adipogenesis in BMSCs, while GluOC alleviated this phenomenon. In addition, high glucose enhanced TP63 expression while GluOC diminished it. Knock-down of TP63 by siRNA transfection restored the inhibitory effect of high glucose on osteogenic differentiation. Furthermore, we detected the downstream signaling pathway PTEN/Akt/GSK3β. We found that diminishing TP63 decreased PTEN expression and promoted the phosphorylation of Akt and GSK3β. We then applied the activator and inhibitor of Akt, and concluded that PTEN/Akt/GSK3β participated in regulating the differentiation of BMSCs.

**Conclusions:**

Our results indicate that GluOC reduces the inhibitory effect of high glucose on osteoblast differentiation by regulating the TP63/PTEN/Akt/GSK3β pathway. TP63 is a potential novel target for the prevention and treatment of diabetic osteoporosis.

**Supplementary Information:**

The online version contains supplementary material available at 10.1186/s12860-021-00365-7.

## Background

According to the World Health Organization (WHO), the number of people with diabetes rose from 108 million in 1980 to 422 million in 2014 [1]. Osteoporosis is one of the several potential long-term complications of diabetes mellitus [2]. The severity of diabetic complications is usually proportional to the degree of hyperglycemia [3]. Deng et al. cultured BMSCs in sera obtained from human subjects with different levels of glycemia; they found that all the hyperglycemic sera inhibited osteoblastic differentiation of BMSCs [4]. BMSCs from the bone marrow can differentiate into osteoblasts and adipocytes and play a critical role in bone homeostasis [5]. Therefore, it is important to explore the potential mechanism of high glucose level inhibition on osteogenic differentiation of BMSCs and identify relevant targets for the treatment of diabetes-induced osteoporosis.

Osteocalcin, which is secreted by mature osteoblasts, is not only embedded in the bone matrix to maintain the bone strength, but also functions like a protein hormone after decarboxylation [6]. Decarboxylation of γ-carboxylated osteocalcin in position 17, 21, 24 is referred to as uncarboxylated osteocalcin (also known as GluOC) [7]. GluOC can regulate insulin metabolism by promoting the secretion of insulin from the islet cells [8]. Moreover, knock-down of osteocalcin in human MSCs causes delayed bone mineralization and decreases the expressions of osteoblast genes [9]. Our laboratory has demonstrated that addition of GluOC in vitro mitigated the damage caused by high glucose in mouse pre-osteoblast cell line MC3T3-E1 [10]. GluOC has been shown to promote osteogenic differentiation of BMSCs [11]. However, the underlying molecular mechanism of the effects of GluOC treatment of BMSCs under high glucose condition still remains obscure.

TP63, one of the p53 family members, is gradually attracting the attention of researchers due to its important role in epidermal morphogenesis [12]. Moreover, TP63 was found to play a critical role in regulating energy metabolism, by increasing fatty acid synthesis and decreasing fatty acid oxidation; it was also shown to play an important role in controlling blood glucose levels especially after the addition of metformin [13]. TP63 is involved in many signaling pathways. Recent research has demonstrated that knockdown of TP63 in normal liver cells of mice under high glucose conditions led to a decrease in PTEN, which increased the phosphorylation levels of Akt and GSK3β and promoted glycogen synthesis in liver cells [14]. Notably, Zhu et al. reported that the effect of high glucose in diminishing cell cycle progression of BMSCs is mediated via PTEN upregulation and Akt inactivation [15]. GSK3β not only plays an important role in glucose metabolism in cells, but also acts as an intermediate molecule in the Wnt/β-catenin signaling pathway, which directly promotes RUNX2 [16]. Decreasing the ninth Serine residue phosphorylation of GSK3β led to increased β-catenin phosphorylation level [17], which is adverse to its survival and nuclear transportation [18]. However, the signaling pathways related to TP63 and BMSCs osteogenic differentiation need to be clarified.

In this study, we sought to investigate the mechanism by which GluOC alleviated high glucose-induced suppression of osteogenic differentiation of BMSCs.

## Results

### GluOC promoted osteogenesis and inhibited adipogenesis in BMSCs under high-glucose condition

**Fig. 1:**
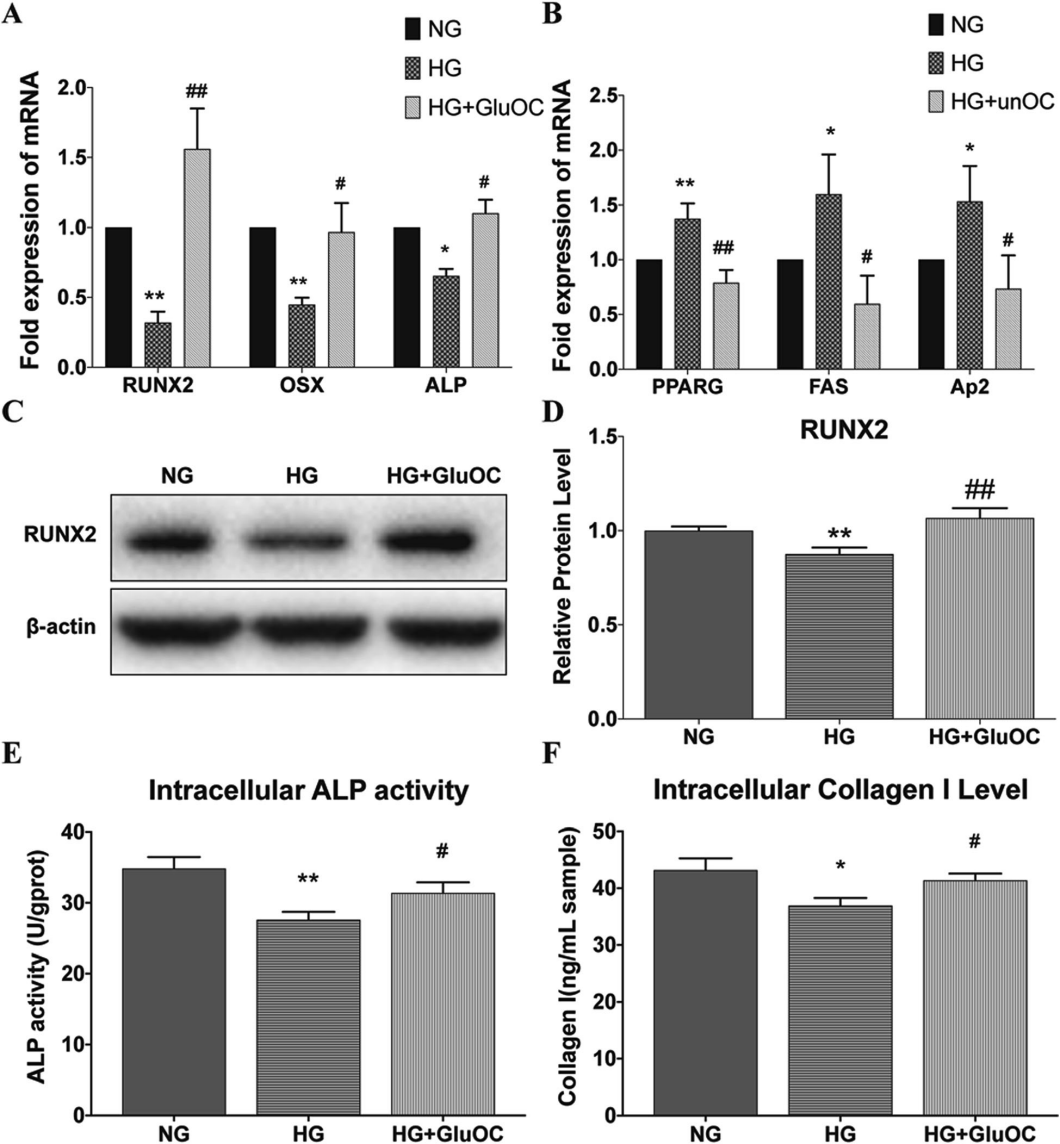
Effects of HG or GluOC on BMSCs. Results of the three treatment groups are shown: normal group (NG; glucose 5.5 mM), high glucose group (HG; glucose 25.5 mM), high glucose plus uncarboxylated osteocalcin group (HG + GluOC; glucose 25.5 mm and GluOC 3 ng/mL). **a** Relative mRNA expression levels of osteogenic genes (RUNX2, OSX, ALP); **b** relative mRNA expression levels of lipogenic genes (PPARγ, Ap2, FAS). **c** Western Blot images of RUNX2 and β-actin are shown; the relative protein expression level is calculated according to the gray value. **d** Intracellular ALP activity and (**e**) Collagen I level. *P < 0.05, **P < 0.01 versus NG; ^#^*P* < 0.05, ^##^*P* < 0.01 versus HG

To examine the effect of GluOC on BMSCs in high glucose conditions, we cultured the BMSCs for 3 days in normal glucose medium (5.5 mM), high glucose medium (25.5 mM), and high glucose medium with GluOC (3 ng/mL). Subsequently, we performed qRT-PCR to characterize the expression of genes associated with osteogenesis (OSX, RUNX2, ALP) and adipogenesis (FAS, PPARγ, Ap2) in BMSCs (Figs. 1a, b). We also detected the protein level of RUNX2 (Fig. 1c, d), which matched the changes in mRNA. The results revealed that high glucose increased the adipogenesis mRNA and decreased the osteogenesis mRNA, while GluOC treatment reversed these changes. The ALP activity was obviously decreased in the high-glucose group (Fig. 1e); however, GluOC increased the activity of this enzyme. In addition to these tests, we also assessed the expression of type I collagen (Col I) in the cells (Fig. 1f). As one of the signs of osteogenesis, the expression of type I collagen also conformed to the previous trend. High glucose reduced the expression of type I collagen while addition of GluOC restored it. These results indicated that GluOC alleviated high glucose-induced suppression of osteogenic differentiation of BMSCs.

### GluOC alleviated high glucose-induced suppression of osteogenic differentiation of BMSCs by TP63

#### High glucose increased TP63 while GluOC had the opposite effect

**Fig. 2:**
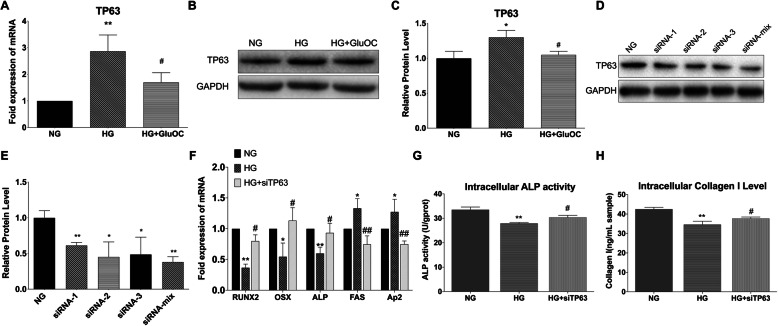
Knock-down of TP63 promoted osteogenesis. **a** mRNA expressions of TP63 in BMSCs in different groups after treatment for 3 days. **b**, **c** Western Blot analysis of TP63 expression in BMSCs in different groups after treatment for 5 days. **d**, **e** Results of WB showing the effect of different siRNAs on the protein level of TP63 in cells under normal glucose treatment; siRNA-mix implies a mixture of siRNAs 1, 2, and 3 in the same proportion. **f**, **g**, **h** mRNA expressions of osteogenic and adipogenic genes in cells, ALP enzyme activity, and collagen I expression, respectively. The treatment groups were normal glucose (NG), high glucose (HG), and high glucose plus siTP63 (HG + siTP63). Negative control siRNA was used in all the RNA interference experiments. *P < 0.05, **P < 0.01 versus NG; ^#^*P* < 0.05, ^##^*P* < 0.01 versus HG

To verify the effect of high glucose and GluOC on TP63, we treated cells in different media (normal glucose, high glucose, and high glucose plus GluOC) for 3 days and measured mRNA and protein expressions of TP63. The results showed that high glucose indeed activated TP63; these effects were reversed by treatment with GluOC (Figs. 2a, b and c).

#### Knock-down of TP63 restored osteogenic inhibition caused by high glucose

To characterize the role of TP63 in osteogenic differentiation, we tested the designed siRNAs including siRNA-mix, which was a mixture of siRNA-1, siRNA-2, and siRNA-3 in a ratio of 1:1:1 (Fig. 2d). siRNA-mix had the best effect in knocking down TP63. Subsequently, we examined the effects of siRNA-mix of TP63 (hereafter abbreviated as siTP63) on the expressions of osteogenic and adipogenic genes in cells cultured in high glucose medium (Fig. 2e). High glucose decreased RUNX2, OSX, ALP, and increased Ap2 and FAS; knock-down of TP63 restored the expressions to normal level (Fig. 2f). Furthermore, we tested ALP activity and Col I level (Figs. 2g and h); the results revealed that high glucose decreased ALP activity and Col I protein levels, while knock-down of TP63 reversed these effects, similar to the effect of GluOC. Collectively, these findings indicated that high glucose inhibited osteogenesis only in the presence of TP63.

### GluOC decreased TP63 to influence PTEN/Akt/GSK3β pathway and promoted osteogenesis and inhibited adipogenesis in BMSCs under high-glucose condition

#### GluOC decreased TP63 to influence PTEN/Akt/GSK3β

**Fig. 3:**
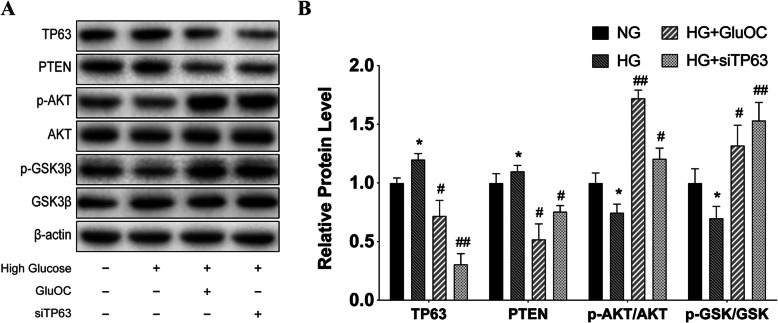
Protein levels of PTEN/Akt/GSK3β. **a** Expressions of various proteins in the BMSCs in the four treatment groups [normal glucose (NG), high glucose (HG), high glucose plus uncarboxylated osteocalcin (HG + GluOC), and high glucose plus siTP63 (HG + siTP63)]. **b** Relative protein expressions obtained by grayscale scanning. Negative control siRNA was used in all the RNA interference experiments. *P < 0.05, **P < 0.01 versus NG; ^#^*P* < 0.05, ^##^*P* < 0.01 versus HG

After confirming the important role of TP63 in osteogenic differentiation, we sought to analyze the pathways and downstream proteins that mediated the effect of TP63. Therefore, we verified these results in BMSCs (Figs. 3a, b). The cells were divided into normal group, high glucose group, high glucose with siTP63 group, and high glucose plus GluOC group. TP63, PTEN, p-Akt, p-GSK3β, and β-catenin levels were detected in the cells. The results showed that high glucose promoted TP63 and increased PTEN expression. We also observed that high glucose inhibited p-Akt levels and decreased GSK3β phosphorylation levels. Application of siTP63 decreased TP63, diminished PTEN, and promoted the phosphorylation of Akt and GSK3β. This result was consistent with the effect of GluOC. These results indicated that TP63 was the upstream regulator of PTEN/Akt/GSK3β.

#### PTEN/Akt/GSK3β participated in regulating the differentiation of BMSCs

**Fig. 4:**
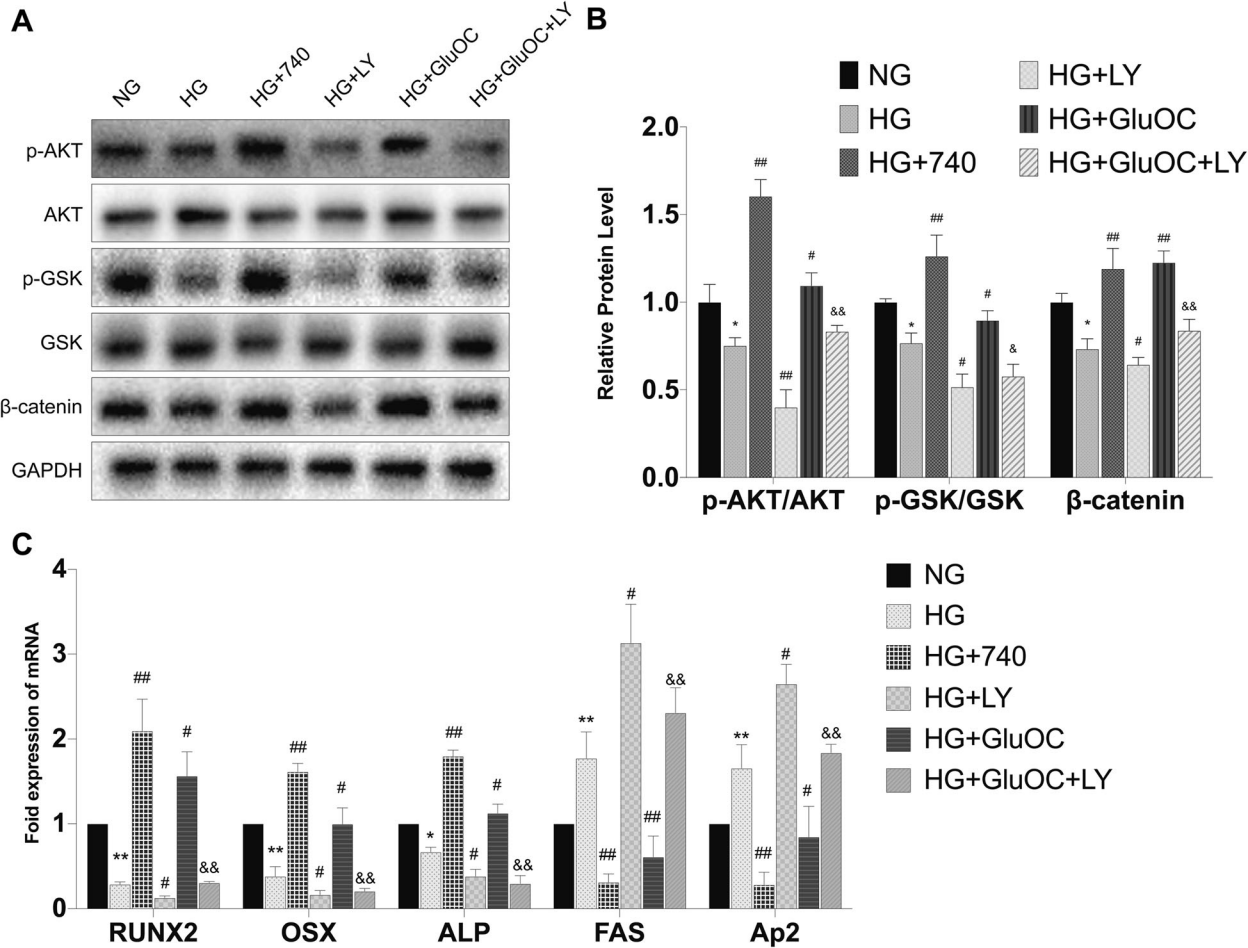
Akt inhibition suppressed osteogenesis while Akt activation promoted osteogenesis. **a** and **b** Relative protein levels of Akt, GSK3β, and β-catenin in BMSCs after different treatments including HG, 740Y-P, LY294002, and GluOC. **c** Expressions of osteogenic genes RUNX2, OSX, ALP, and adipogenic genes FAS and Ap2. *P < 0.05, **P < 0.01 versus NG; ^#^*P* < 0.05, ^##^*P* < 0.01 versus HG; ^&^*P* < 0.05, ^&&^*P* < 0.01 versus HG + GluOC

We next probed the impact of the PTEN/Akt/GSK3β pathway on osteogenesis by using 740Y-P (25 μM) or LY294002 (10 μM). 740Y-P is the activator of Akt and LY294002 is the inhibitor of Akt. High glucose decreased the phosphorylation of Akt and GSK3β, similar to β-catenin (Figs. 4a, b). Treatment with 740Y-P increased the phosphorylation of Akt and GSK3β. In the next group, application of LY294002 in the medium caused even greater inhibition of Akt phosphorylation; in addition, there was decrease in the phosphorylation of GSK3β and protein level of β-catenin. The other two groups were designed to investigate whether LY294002 can inhibit the effect of GluOC. GluOC increased the expressions of p-Akt, p-GSK3β, and β-catenin; however, after addition of LY294002, all these expressions decreased. As shown in Figs. 4a and b, GSK3β was regulated by PTEN/Akt. Figure 4c showed the expressions of osteogenic and adipogenic genes. 740Y-P increased osteogenesis and decreased adipogenesis, while LY294002 had the opposite effect. In addition, after pre-treatment of cells with LY294002, GluOC was not able to increase osteogenesis and decrease adipogenesis under high glucose conditions. These results indicated that PTEN/Akt/GSK3β was associated with the differentiation of BMSCs.

## Discussion

In this study, we demonstrated for the first time that GluOC alleviates high glucose-induced suppression of osteogenic differentiation of BMSCs by inhibiting TP63. Decreasing the expression of TP63 inhibited PTEN which upregulated the phosphorylation of Akt and GSK3β. Consequently, the osteogenic genes were promoted and adipogenic genes were inhibited.

Diabetes has an adverse effect on bone development and remodeling. In a study of 33,000 middle-aged people, diabetes was found to be the strongest predictor of low-energy fractures in both men and women (relative risk: 2.38 and 1.87, respectively) [19]. In the high-glucose mouse model, HE and Masson staining of bone tissue showed inhibition of bone regeneration compared to negative control; in addition, protein expressions of ALP and RUNX2 were diminished [20]. In human MSCs, high glucose not only reduced cell viability and increased the number of β-galactosidase-positive cells, but also suppressed osteogenic differentiation [21]. Previous research at our laboratory indicated that high glucose promotes adipogenic differentiation and suppresses osteogenic differentiation of MG-63 cells by stimulating the cAMP/PKA/ERK pathway [22]. Besides, we demonstrated the involvement of oxidative stress in the process of high glucose-induced inhibition of osteogenesis and promotion of adipogenesis through the PI3K/Akt pathway in rat primary osteoblasts [23]. In this study, we found that high glucose inhibited osteogenesis of BMSCs and promoted adipogenesis. Therefore, restoring the inhibition of osteogenic differentiation caused by hyperglycemia is a potential strategy for treatment of diabetic osteoporosis.

As an effective insulin secretagogue, GluOC regulates the glucose and lipid metabolism by targeting various tissues and organs such as adipocytes, small intestine, and skeletal muscle [6]. Osteocalcin knockout mice showed abnormal visceral fat accumulation and severely impaired glucose metabolism [8]. Preliminary laboratory studies have demonstrated that GluOC promotes osteogenic differentiation under hyperglycemic conditions. GluOC was shown to impede high glucose-induced reactive oxygen species (ROS) production and to promote osteoblast differentiation in MC3T3-E1 cells under high glucose conditions [10]. We also found that GluOC promotes osteoblastic differentiation of BMSCs through the Erk-Smad/β-catenin pathway under normal glucose conditions [11]. In this study, GluOC restored high glucose-induced inhibition of osteogenic differentiation of BMSCs. However, the underlying molecular mechanisms are still obscure.

TP63 is a transcription factor that participates in glucose and lipid metabolism. TP63 knockout mice showed insulin resistance, obesity, and glucose intolerance; TP63 prevented these symptoms by increasing fatty acid synthesis and reducing fatty acid oxidation through the SIRT1/AMPKα2/LKB1 pathway [13]. The important role of TP63 in bone development is even more noteworthy. Kawata et al. generated AER (the apical ectodermal ridge)-specific TP63 knock out mice procreated by mice with a null and a flox allele of TP63 and mice with Msx-Cre [24]. The neonates showed limb malformation, which indicated that TP63 is essential for limb bud during embryonic development. Lu et al. transfected MSCs with shRNA to decrease TP63, which prevented chemotherapeutic drug-induced apoptosis of MSCs [25]. In this study, high glucose increased the expression of TP63 in BMSCs while GluOC decreased it. We showed that knockdown of TP63 under high glucose conditions restored osteogenic differentiation and inhibited adipogenic differentiation of BMSCs, as manifested by the increased expressions of osteogenic genes, decreased expressions of adipogenic genes, and upregulation of ALP activity and COLI production. As an upstream regulator of BMSCs differentiation, TP63 does play an important role. However, the regulation of differentiation in vivo is possibly more complicated. There might be more upstream regulatory factors involved in the process waiting for us to discover.

We also showed that knock-down of TP63 inhibited PTEN expression and promoted the phosphorylation of Akt and GSK3β. PTEN is a negative regulator of PI3K/Akt, which is the main signal pathway of cell growth and survival [26, 27]. Liu et al. used celecoxib to upregulate PTEN in human BMSCs, which induced decline in the expression of RUNX2; however, treatment with SF1670, an inhibitor of PTEN, promoted the expression of RUNX2 [28]. We added inhibitors and agonists to detect the effect of Akt on the downstream proteins and genes. As depicted in Fig. 4, activation of Akt promoted GSK3β phosphorylation, β-catenin relative protein content, and osteogenic gene expression, while suppression of Akt had the opposite effect. GSK3β/β-catenin are downstream of Wnt signaling pathway, activation of which is beneficial for bone development [29]. Taken together, TP63 was found to affect the osteogenic differentiation of BMSCs via the PTEN/Akt/GSK3β signaling pathway, and GluOC bolstered high glucose-induced suppression of osteogenic differentiation of BMSCs via the TP63/PTEN/Akt/GSK3β.

## Conclusions

**Fig. 5:**
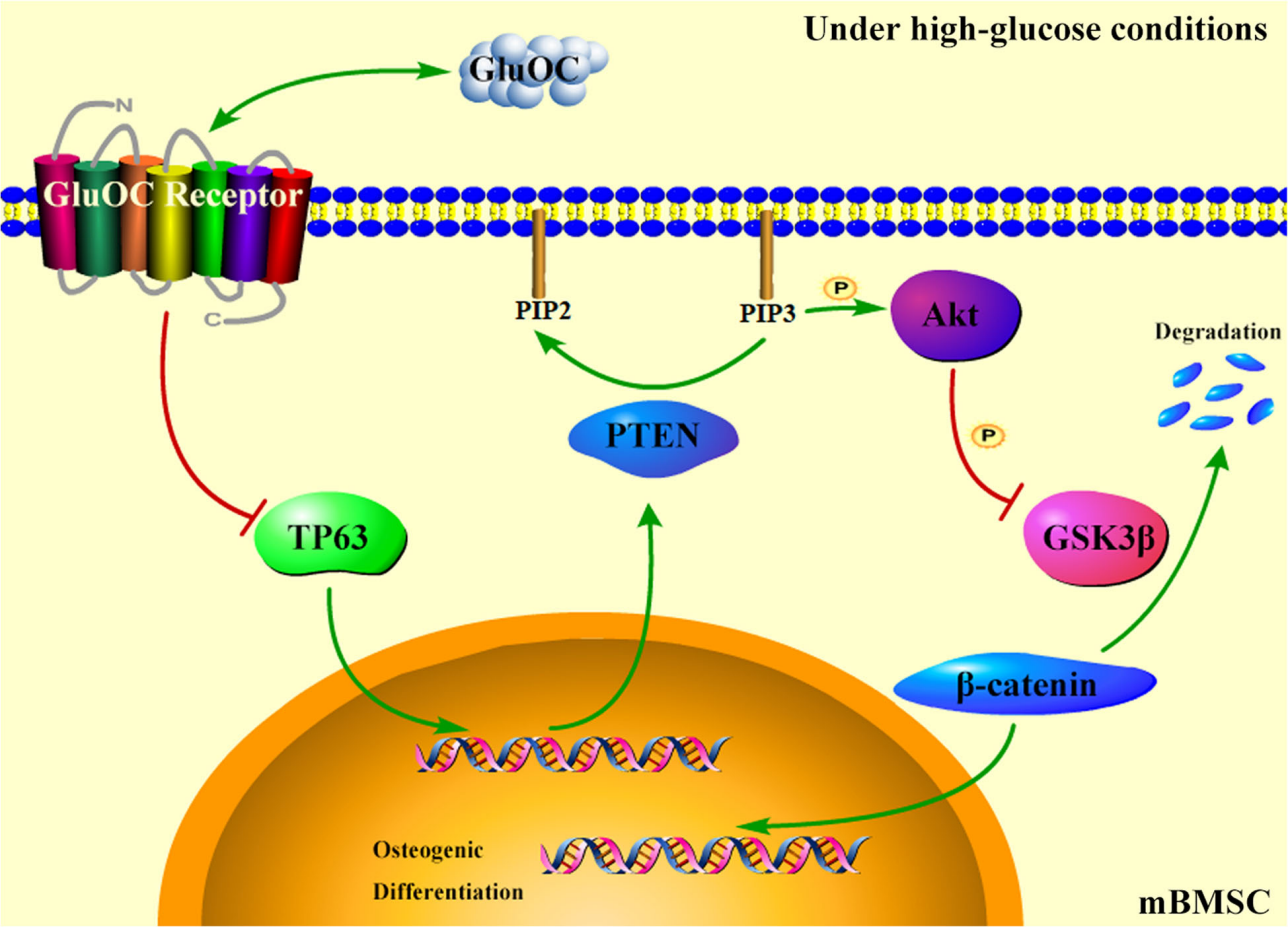
Schematic drawing of the signaling pathway

In this research, we established that TP63 modulates the process by which GluOC regulates high glucose-induced suppression of osteogenic differentiation of BMSCs. GluOC alleviated the inhibitory effects of high glucose on the osteogenic differentiation of BMSCs. Additionally, GluOC inhibited TP63 and PTEN, increased phosphorylation of Akt and GSK3β, and finally promoted the expressions of osteogenic genes and inhibited those of adipogenic genes in BMSCs under high glucose conditions (Fig. 5). In conclusion, we not only explained the mechanism of GluOC more clearly, but also provided a new target (TP63) for the prevention and treatment of diabetic osteoporosis.

## Methods

### Materials

Four-week-old male C57BL/6 mice (certificate number SCXK 2018–0008) were purchased from the Beijing Vital River Laboratory Animal Technology Co. Ltd. (Beijing, China). The basal medium was composed of 89% α-MEM (Hyclone, USA), 10% fetal bovine serum (SeraPro, Germany), and 1% penicillin and streptomycin (Hyclone). Total RNA Extraction Kit, One-Step gDNA Removal and cDNA Synthesis SuperMix, and Top Green qPCR SuperMix (+Dye II) were purchased from TransGen Biotech, China. RIPA lysis buffer (Beyotime Biotechology, China), phenylmethylsulfonyl fluoride (PMSF; Gibco, USA), and BCA Protein Assay Kit (Lablead, China) were used for Western blot.

### BMSCs culture

The animal study was approved by the local committee for Animal Care and Ethics. The procedures used in this study conform to the tenets of the Declaration of Helsinki. Mice were sacrificed and their bilateral tibia and femur bones were stripped from the muscle tissue. After washing the bones repeatedly with PBS, the bone marrow was flushed out of the bone with basal medium. Next, we incubated the bone marrow in 10-cm petri dishes for 72 h at 37 °C and 5% CO_2_. Finally, the fibroblast-like cells adhering to the dishes were BMSCs. The morphological observation, osteogenic differentiation and adipogenic differentiation capacity of BMSCs are described in Supplementary 1. All animal experiments followed the Guide for Care and Use of Laboratory Animals in China. The basal medium was changed every 48 h. After attaining 90% confluence, the cells were digested with Trypsin-EDTA solution (Beyotime, China) and passaged. Cells from the third or fourth generation were used in the subsequent experiments.

### Preparation of GluOC

As described elsewhere [30], we constructed recombinant mouse GluOC with six-histidine tag. Next, we used DE3 (TransGen Biotech, China) to express the reconstructed GluOC. We then collected the expressed proteins which had been purified after passing through a Ni Sepharose™ 6 Fast Flow column (GE healthcare, Sweden). After using Coomassie blue staining to detect the purity of the protein, osteocalcin ELISA (Immunotopics, San Clemente, USA) was used to detect the protein concentration.

### Real-time qPCR

In order to detect the effect of different treatments on the expressions of different genes in BMSCs, we first extracted the total RNA of the cells. Next, the total RNA was reverse transcribed to cDNA according to the manufacturer’s instructions. Then we added the cDNA as a template to the system for qPCR. Glyceraldehyde-3-phosphate dehydrogenase (GAPDH) served as the endogenous normalization control, and the fold change of different test genes were determined using the ΔΔCT method. The sequences of different genes’ primers are listed in Table 1. All primers were synthesized by Sangon Biotech (Shanghai) Co. Ltd.

**Table 1:** The primer sequences

Gene	Primer sequences(5′ → 3′)	Accession Number
RUNX2	F: GCCGGGAATGATGAGAACTA R: GGACCGTCCACTGTCACTTT	NM_001146038.2
OSX	F: CTAGTTCCTATGCTCCGACC R: TCATCACATCATCATCGTG	NM_130458.1
ALP	F: CAAAGGCTTCTTCTTGCTGGT R: AAGGGCTTCTTGTCCGTGTC	NM_130458.1
FAS	F: TGCTTGCTGGCTCACAGTTAAGAG R: TCAGGTTGGCATGGTTGACAGC	NM_007988.3
Ap2	F: AAGTGGGAGTGGGCTTTG R: GTCGTCTGCGGTGATTTC	NM_024406.3
TP63	F: CTGGAAAACAATGCCCAGAC R: GAGGAGCCGTTCTGAATCTG	NM_001127262.1
GAPDH	F: ACCCAGAAGACTGTGGATGG R: CACATTGGGGGTAGGAACAC	NM_130458.1

### Immunoblotting

All the BMSCs lysates were acquired by adding RIPA lysis buffer, phenylmethylsulfonyl fluoride. Protein concentrations were determined using the BCA Protein Assay kit. We used 10% or 8% sodium dodecyl sulfate-polyacrylamide gel electrophoresis (SDS-PAGE) to separate approximately 20 μg of protein extracts, and then transferred them to a polyvinylidene fluoride (PVDF) membrane. After blocking the PVDF with milk, we incubated it overnight at 4 °C with the primary antibody. PVDF was washed three times with TBST, and then incubated with goat anti-rabbit antibody for 1 hour, followed by its exposure. Primary antibodies (Rabbit anti Mouse) of RUNX2, p-GSK3β^ser9^, β-catenin were acquired from Cell Signaling Technology, USA; Akt, p-Akt^ser473^, TP63, PTEN, GSK3β from Abcam, UK; β-actin and GAPDH from Cohesion Biosciences, UK. The secondary antibodies (Goat anti Rabbit, HRP) were acquired from Lablead, China.

### Enzyme-linked immunosorbent assay for type I collagen

Cells were seeded equally in 6-well dishes. After attainment of 70% confluence, the basal media were changed to media containing different treatments for 5 days. The level of secreted Col I was determined by ELISA Kit for Col I (Cloud-Clone Corp., USA) according to the manufacturer’s instructions.

### Alkaline phosphatase (ALP) assay

Cells seeded in 6-well dishes were cultured in different media for 5 days. According to the manufacturer’s instructions, the intracellular ALP activity was measured using an ALP assay kit (Nanjing Jiancheng Institute of Bioengineering, China).

### Inhibition of TP63 by RNA interference

Interfering fragments were designed and produced by GenePharma, China. The siRNA fragments were mixed with Lipofectamine 3000 (Thermo Fisher Scientific, USA) in a solution of α-MEM. After incubation for 20 min, the solution was added to cell medium which were changed to serum-free medium. At 6 h, we changed the medium to basal medium or other different treatments. After 48 h, cells were collected for testing. The siRNA sequences were as follows--siRNA1 sense: 5′-CCCUC-AGCAC-ACGAU-CGAAT-T-3′; siRNA2 sense: 5′-GACGC-AUUGU-CAGUU-UCUUT-T-3′; siRNA3 sense: 5′-CCCUG-AACAG-UUCCG-ACAUT-T-3′; Negative control siRNA sense: 5'-UUCUC-CGAAC-GUGUC-ACGUT-T-3'.

### Statistical analysis

All data in this study are expressed as mean ± standard deviation values from 3 independent experiments. Between-group differences were assessed using the *t*-test or one-way ANOVA (ANOVA). All statistical analyses were performed using SPSS (version 22.0; IBM, USA). *P* values < 0.05 were considered indicative of statistical significance.

## Supplementary Information

**Additional file 1: Figure S1.** Identification of BMSCs.

**Additional file 2. **Original images of the immunoblots.

## Data Availability

The datasets used or analysed during the current study are available from the corresponding author on reasonable request.
