# Supplementary material for: Uncarboxylated osteocalcin alleviates the inhibitory effect of high glucose on osteogenic differentiation of mouse bone marrow–derived mesenchymal stem cells by regulating TP63
Source: BMC Mol Cell Biol. 2021 Apr 27;22:24. doi: 10.1186/s12860-021-00365-7 (PMC8080387; doi:10.1186/s12860-021-00365-7)
Supplement: Supplementary file 1 — Additional file 1: Figure S1. Identification of BMSCs. [file 12860_2021_365_MOESM1_ESM.pdf]

Result

After centrifugation of BMSCs, they were put into complete medium and recorded as the first passage (Figure 1A). These cells comprised heterogeneous groups. The initial plating of the adherent cells displayed a small-rounded shape, a spindle shape, or large flattened morphology; there were a large number of round cells floating and failing to adhere to the bottom. In the second passage, they adhered rapidly and expanded. The rounded cells disappeared after consecutive passages and fibroblast-like cells became enriched. In the third passage, the fibroblast-like cells became morphologically homogeneous, and their growth rate increased.

When the cell confluence reached 90%, we added the osteogenic differentiation inducer (ODI) to culture the cells for 15 days, and then stained the cells with Alizarin Red to observe the calcium nodules (Figure 1B and C). And we analyzed the staining results. From the results, the ODI caused the cells to produce significantly more calcium nodules, meaning that the cells successfully differentiated into osteoblasts. Similarly, we added adipogenic differentiation inducer (ADI) and cultured the cells for 12 days, then stained the cells with Oil Red O to observe the formation of lipid droplets (Figure 1D and E). We also analyzed the staining results. The results showed that the inducer caused the cells to produce more lipid droplets, which meant that the cells differentiated into lipocytes. From the perspective of morphology and differentiation potential, these cells are BMSCs.

Method

Osteogenic differentiation induction and Alizarin Red staining

After the cell confluence reached 90%, we discarded the medium and added osteogenic induction medium (ODI; α-MEM with 10% FBS, 5μg/ml insulin, 0.1μM dexamethasone, 0.2mM vitamin C and 10mM β-Glycerol Phosphate). We changed the medium once a day. After we treated the cells for 15

days, we discarded the medium, washed the cells three times with PBS, and fixed the cells with 10% formaldehyde for 30 minutes. We stained the cells with 40mM Alizarin Red for 30min, and then washed them with PBS three times. Finally, we dissolved the dye solution with 0.1M cetylpyridinium chloride on a shaker for 15 minutes, and drew 200μl of the solution to test A570.

Adipogenic differentiation induction and Oil Red O staining

After the cell confluence reached 90%, we discarded the medium and added lipid induction medium (ADI; **α**-MEM with 10% FBS, 10 μg/ml insulin, 0.1 μM dexamethasone, 0.5 mM IBMX, 0.1 mM Indomethacin). We changed the medium once a day. After we treated the cells for 12 days, we discarded the medium, washed the cells three times with PBS, and fixed the cells with 10% formaldehyde for 30 minutes. We stained the cells with 0.6% Oil Red O for 1 hour, and then washed the cells with 75% ethanol three times. Finally, we dissolved the dye solution with isopropanol on a shaker for 10 minutes, and drew 200μl of the solution to test A510.

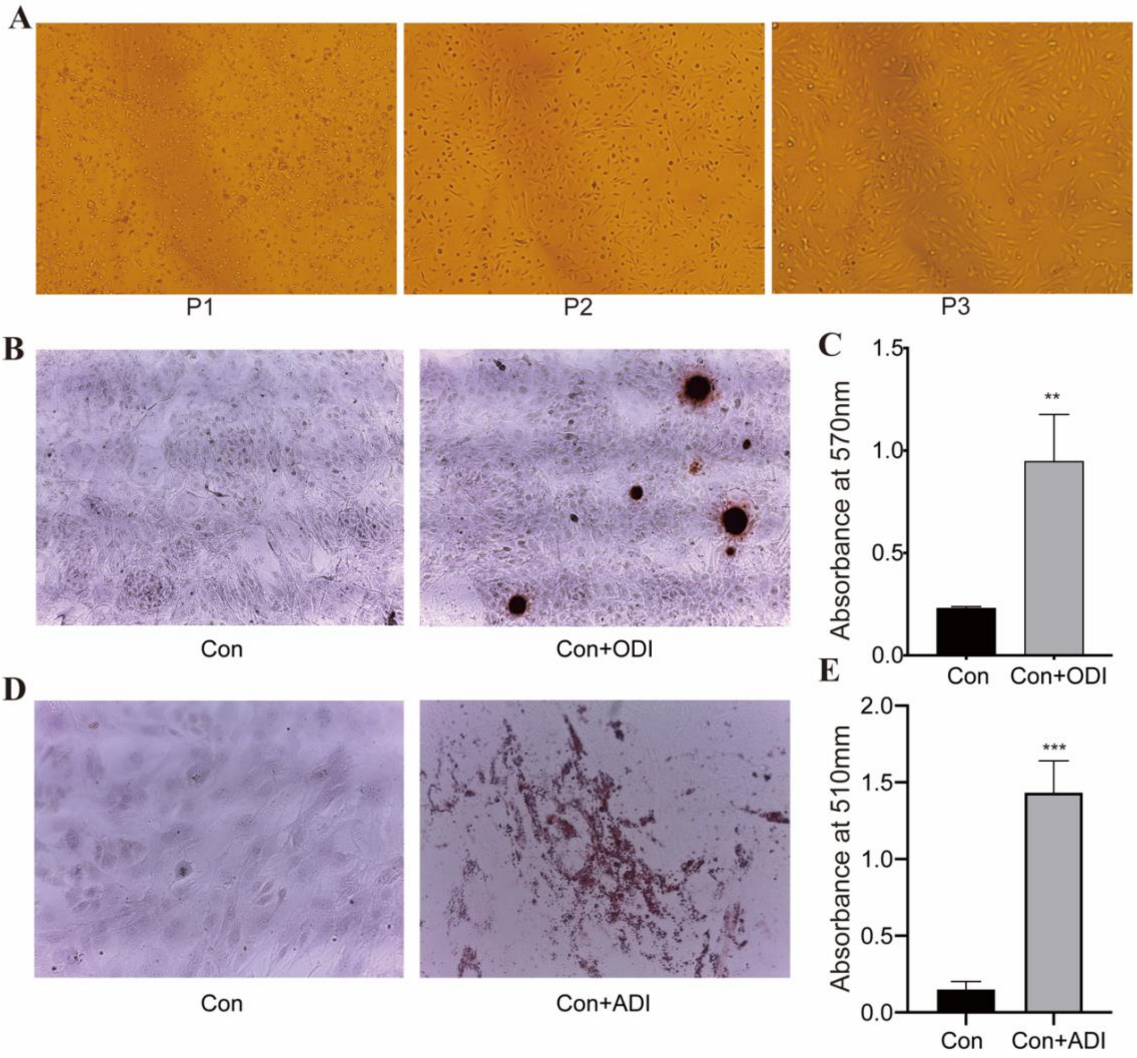

Figure 1. Identification of BMSCs

(A) represents the morphology of BMSCs cells in P1, P2 and P3 generations, respectively. (B) showed the results of alizarin red staining in normal group (Con) and osteoblast differentiation inducer group (Con+ODI). The absorption value of 570nm was detected, and the results were reflected in (C). (D) showed the results of oil red staining in normal group (Con) and adipogenic differentiation inducer group (Con+ADI). The absorption value of 510nm was detected, and the results were reflected in (E). Images of (A) were obtained under a 40x optical microscope; (B) were obtained under a 100x optical microscope; (D) were obtained under a 400x optical microscope. **P <0.01, ***P<0.001 versus Con.
