# Supplementary material for: Uncarboxylated osteocalcin alleviates the inhibitory effect of high glucose on osteogenic differentiation of mouse bone marrow–derived mesenchymal stem cells by regulating TP63
Source: BMC Mol Cell Biol. 2021 Apr 27;22:24. doi: 10.1186/s12860-021-00365-7 (PMC8080387; doi:10.1186/s12860-021-00365-7)

Title:

Uncarboxylated osteocalcin alleviates the inhibitory effect of high glucose on osteogenic differentiation of mouse bone marrow–derived mesenchymal stem cells by regulating TP63

Authors:

Name: Fangzi Gong

Address: Medical School, University of Chinese Academy of Sciences, Beijing, China.

Name: Le Gao

Address: Medical School, University of Chinese Academy of Sciences, Beijing, China.

Name: Luyao Ma

Address: Medical School, University of Chinese Academy of Sciences, Beijing, China.

Name: Guangxin Li

Address: College of sports medicine and physical therapy, Beijing Sport University, Beijing,China.

Corresponding author:

Name: Jianhong Yang

Address: Medical School, University of Chinese Academy of Sciences, Beijing 101400, China.

Email: yangjh@ucas.edu.cn

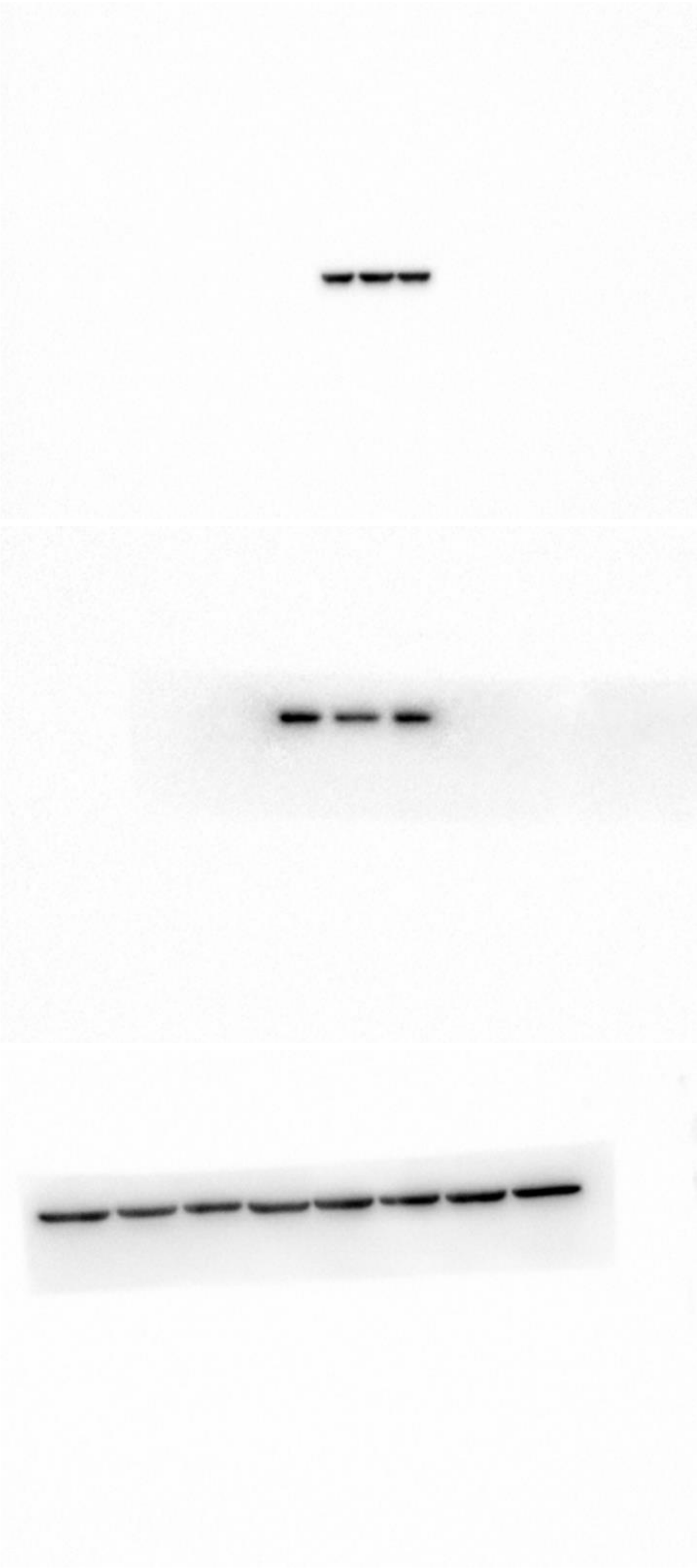

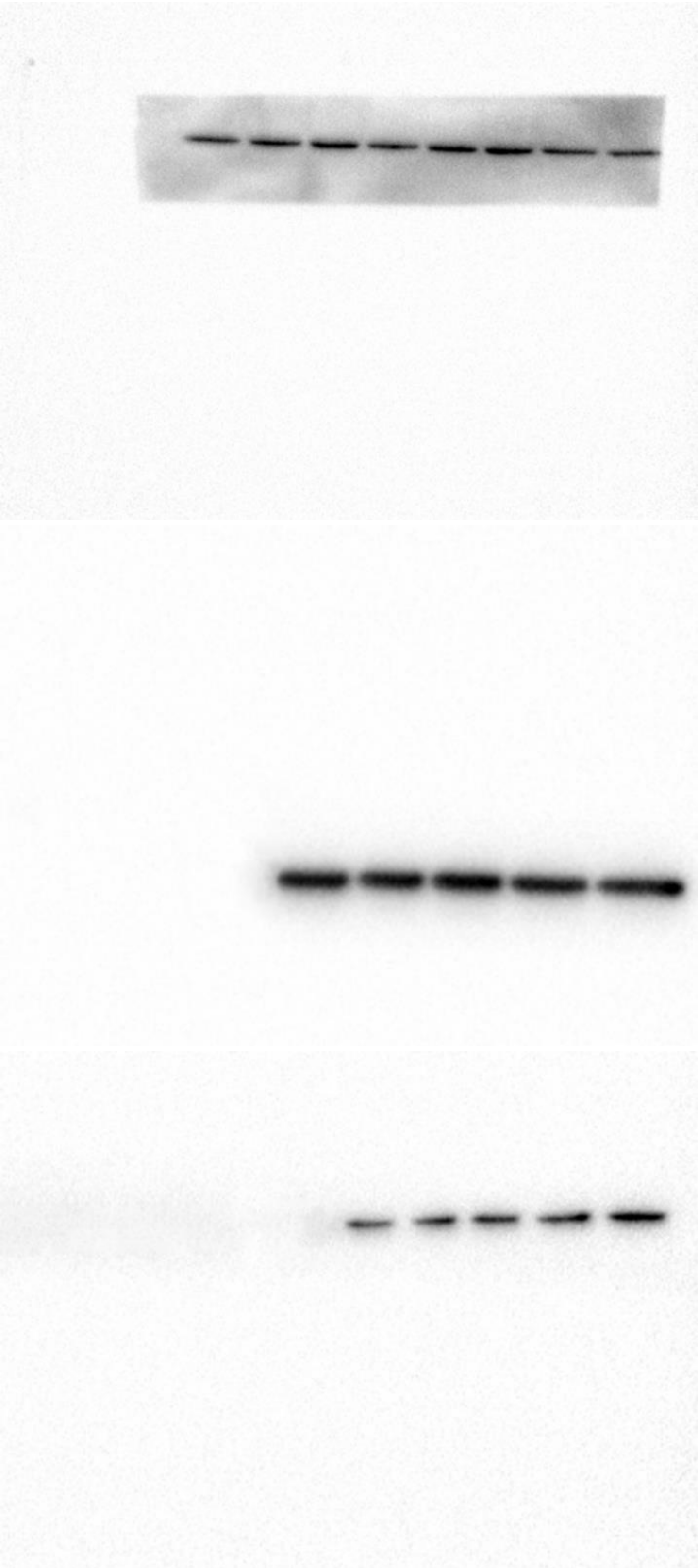

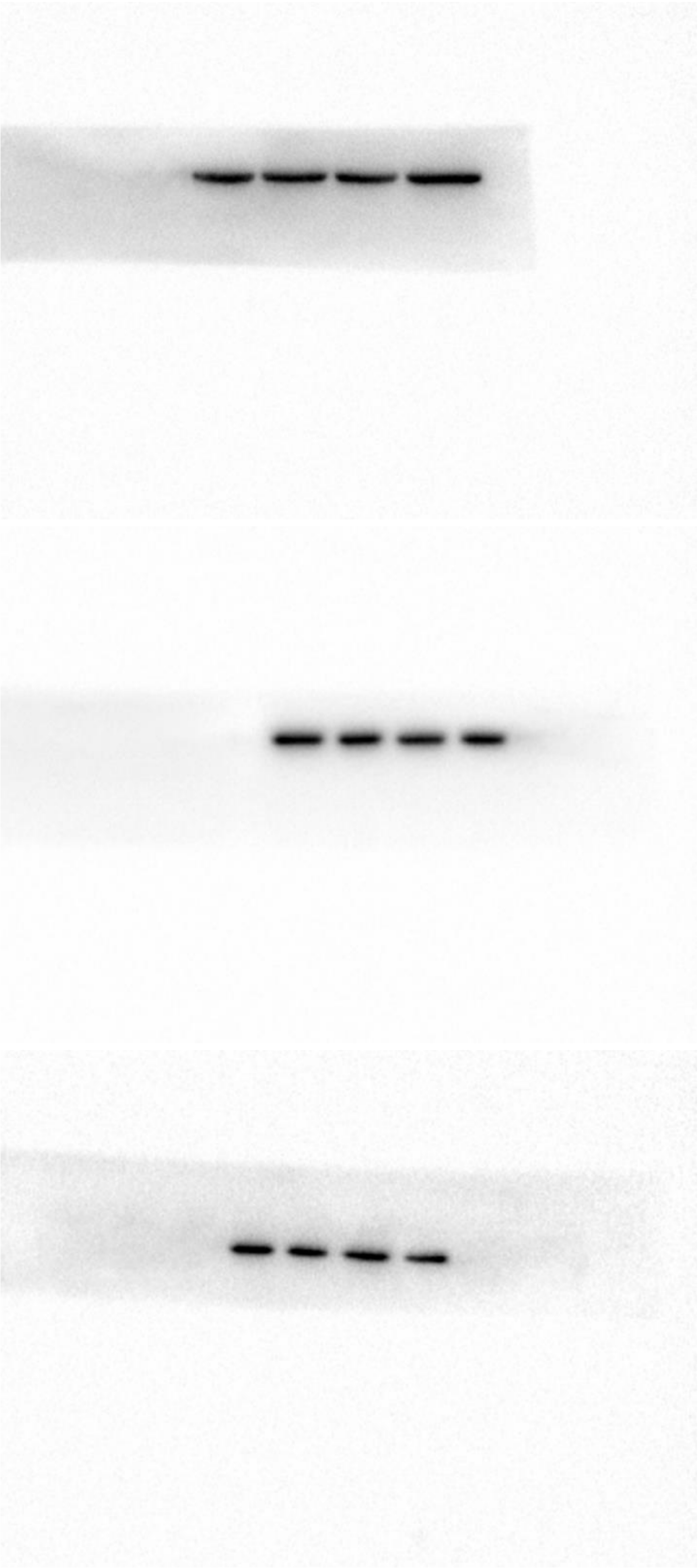

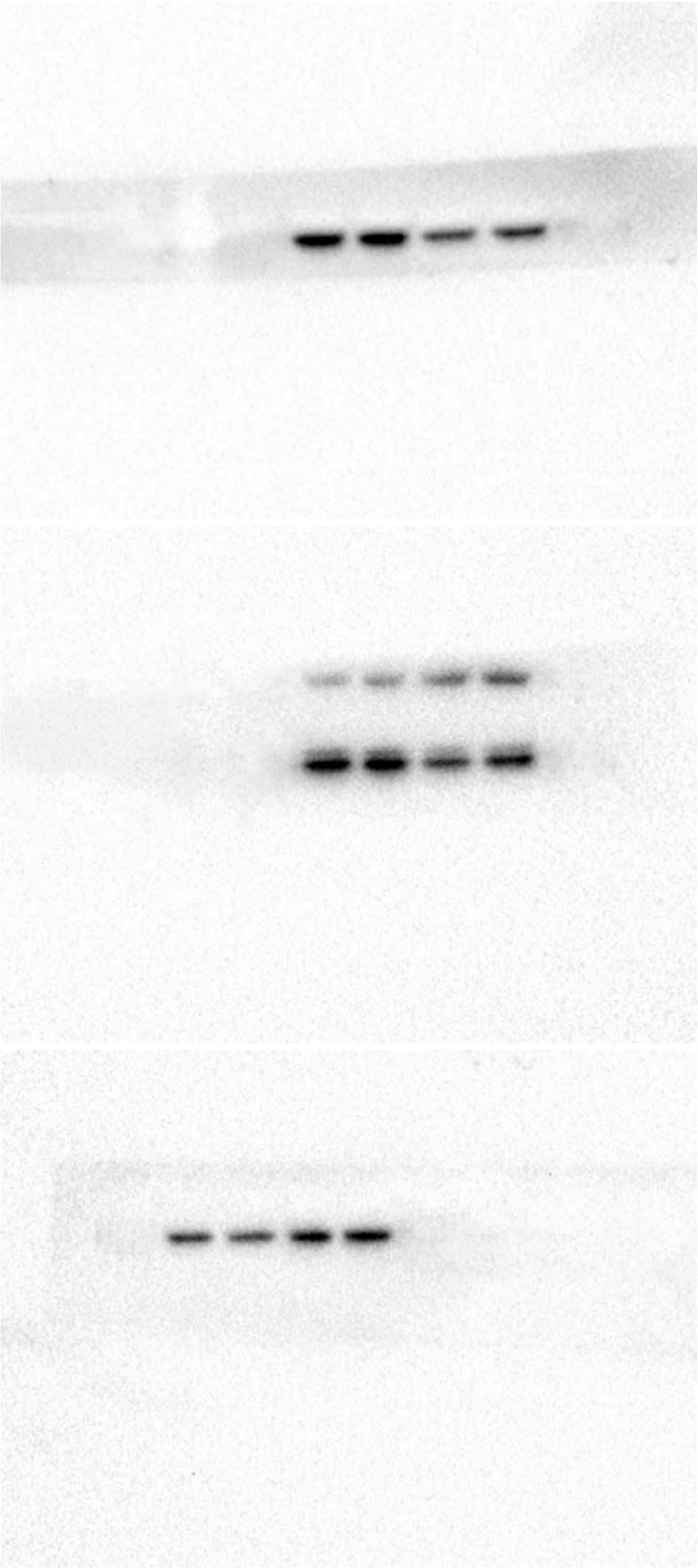

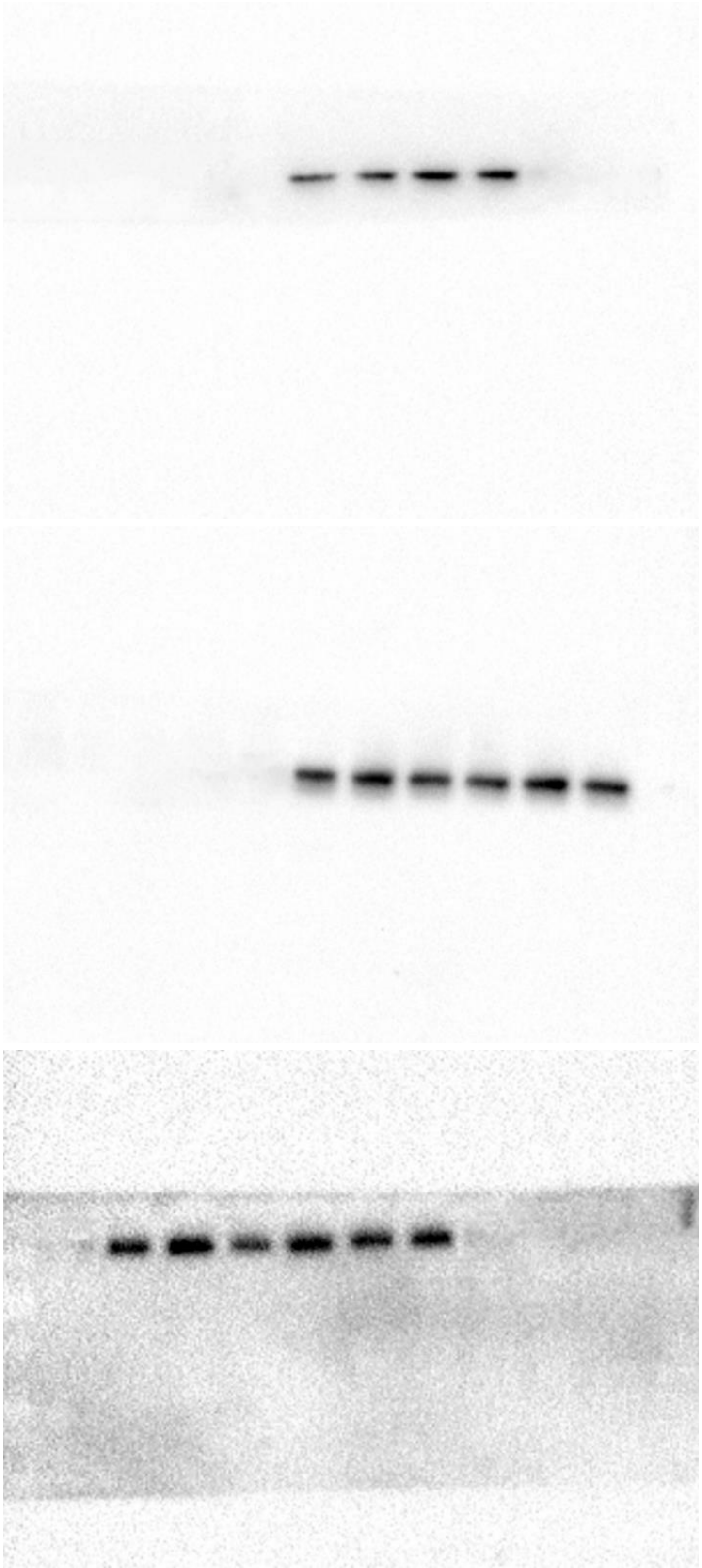

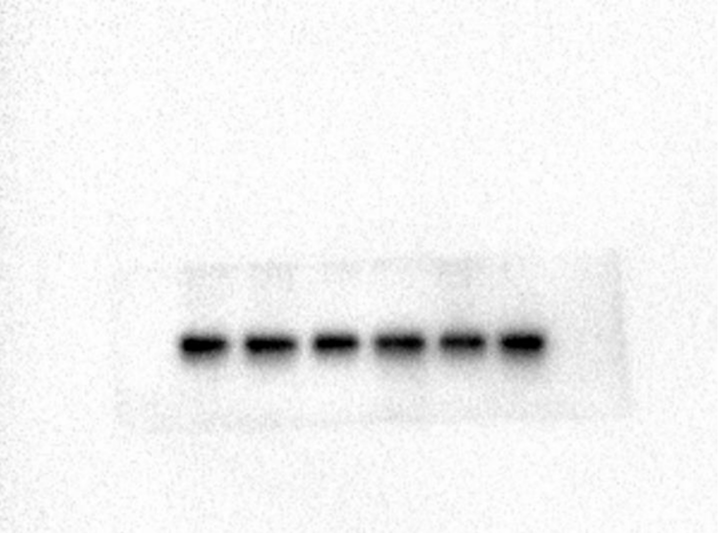

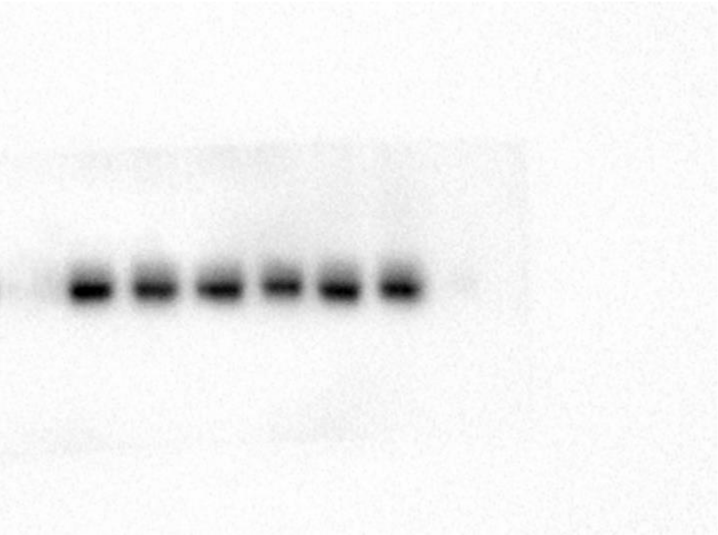

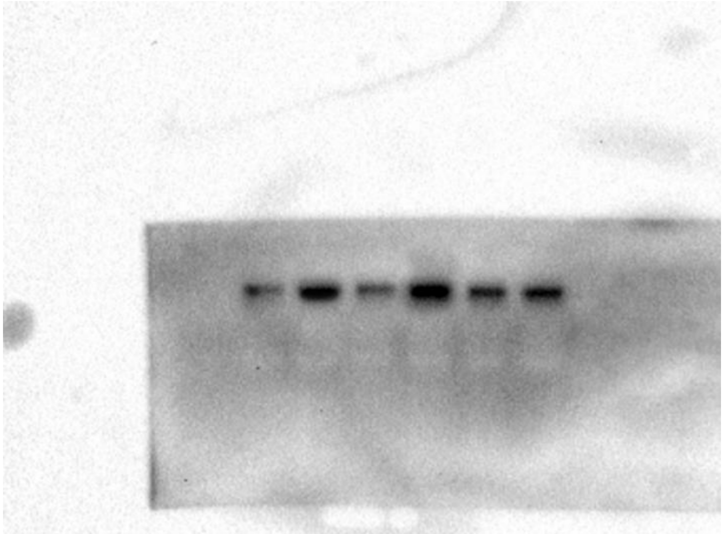

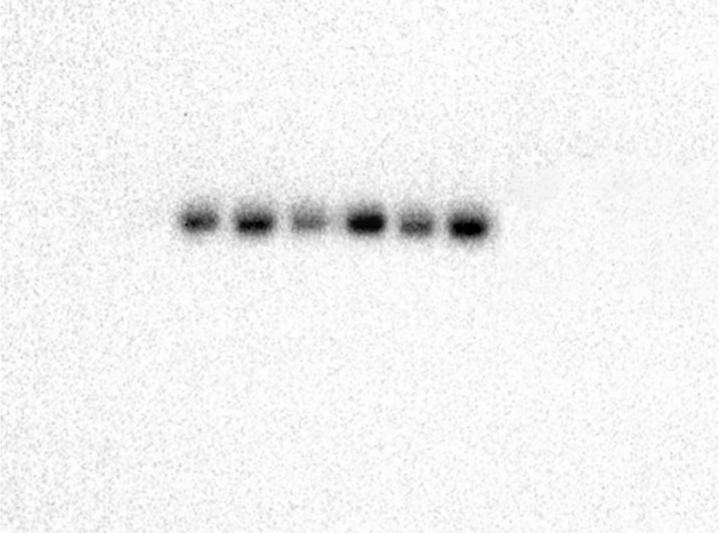

Supplement: Supplementary file 2 — Additional file 2. Original images of the immunoblots. [file 12860_2021_365_MOESM2_ESM.pdf]
